# Fifteen-Year Follow-Up of a Patient with Acinar Cystic Transformation of the Pancreas and Literature Review

**DOI:** 10.1155/2020/8847550

**Published:** 2020-12-26

**Authors:** Xiaoyun Wen, Jela Bandovic

**Affiliations:** Department of Pathology, Stony Brook Medicine, Stony Brook, NY 11794, USA

## Abstract

Acinar cystic transformation (ACT), also known as “acinar cell cystadenoma,” is a rare and newly recognized benign pancreatic cystic neoplasm. However, its true malignant potential remains unknown. Here, we report a case of ACT with 15-year follow-up. A 10-year-old female initially presented with abdominal pain and was found to have a cystic lesion in the region of pancreatic head on computed tomography scan. She underwent an exploratory laparotomy, and the intraoperative biopsy of the cyst wall showed a true pancreatic cyst without malignancy. Her symptoms subsequently resolved, and she was placed under close ultrasound surveillance. For the next fifteen years, the patient was asymptomatic without any complications and had a successful pregnancy. Surveillance showed the tumor grew in size from 4.2 cm to 6.2 cm in diameter. In the latest five months, she noted occasional abdominal pain. A pylorus-preserving pancreaticoduodenectomy was performed. The resected cystic lesion was multilocular and lined by a single layer of bland epithelium ranging from nondescript flat/cuboidal epithelium to apparent acinar cells which were strongly positive for trypsin, so the final diagnosis was confirmed to be ACT. The prior biopsy was retrospectively reviewed to reveal similar epithelial lining. To the best of our knowledge, this is the longest period of follow-up for ACT to date. Our findings suggest that ACT is a slow-growing neoplasm without malignant transformation after fifteen years. Therefore, we recommend biopsy for histologic diagnosis followed by close ultrasound surveillance without surgical intervention in asymptomatic or young ACT patients.

## 1. Introduction

Acinar cystic transformation (ACT), also known as “acinar cell cystadenoma,” is an extremely rare benign pancreatic cystic neoplasm lined by cells with acinar differentiation without cytologic atypia [1]. It was firstly described in April 2002 by Albores-Saavedra as “acinar cystadenoma” in an autopsy case [2] and has been newly recognized as an entity by the World Health Organization (WHO) in 2010. In the literature, a total of 75 patients with ACT of the pancreas have been reported [2–26]. To date, the pathogenesis of ACT still remains unclear but recent evidence suggests that it could be a nonneoplastic dilation of the acinar and ductal epithelium [25]. Although ACT is no longer thought to represent the benign counterpart to acinar cell cystadenocarcinoma, the true malignant potential of ACT has not yet been determined due to the rarity of this entity and relatively short periods of follow-up in the reported cases. Here, we reported a case of ACT with 15-year follow-up. To the best of our knowledge, this is the longest period of follow-up for ACT to date.

## 2. Case Presentation

**Figure 1:**
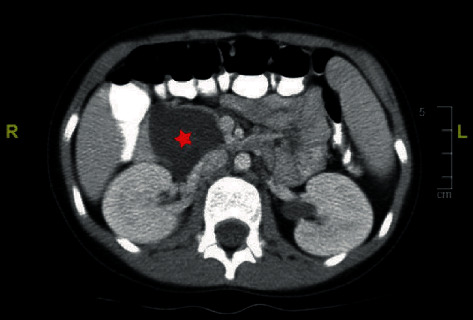
Computed tomographic scan of the abdomen revealed a large cystic lesion (star) in the region of pancreatic head.

A previously healthy 10-year-old female presented to our hospital with a 2-month history of intermittent abdominal pain. An abdominal computed tomography (CT) scan done to rule out appendicitis revealed a 4 × 3 cm cystic lesion in the region of pancreatic head, and whether the lesion involved the pancreas at its head or not was unknown based on the images (Figure 1). The differential diagnoses included a choledochal cyst, a retroperitoneal lymphangioma, and a pancreatic cystic lesion. The patient therefore underwent an exploratory laparotomy to obtain a histologic diagnosis. The intraoperative frozen section diagnosis of the cyst wall biopsy was a true pancreatic cyst without malignancy. The cystic lesion was drained and unroofed. Cystic fluid analysis showed an amylase of 60 U/L. The patient's symptoms subsequently resolved after the procedure, and she was placed under close ultrasound surveillance. The surveillance interval period was 3 months for the first follow-up, 5 months for the second follow-up, 8 months for the third follow-up, and then extended to a year.

For the next fifteen years, the patient was asymptomatic without any complications and had a successful uncomplicated pregnancy. The abdominal ultrasound surveillance showed the tumor grew in size from 4.2 cm to 6.2 cm in diameter for 15 years. In the latest five months, the patient had noted occasional right mid abdominal pain and discomfort. A pylorus-preserving pancreaticoduodenectomy was then performed due to her recurrent pain and enlargement of the lesion.

**Figure 2:**
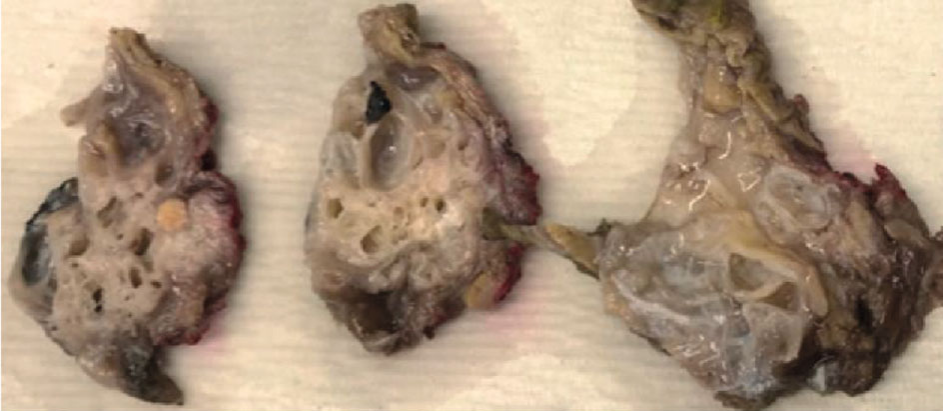
Serial sections of the multilocular cystic lesion in the head of the pancreas showed variable-sized cysts filled with clear fluid, and the cystic wall was smooth without any solid or papillary areas.

**Figure 3:**
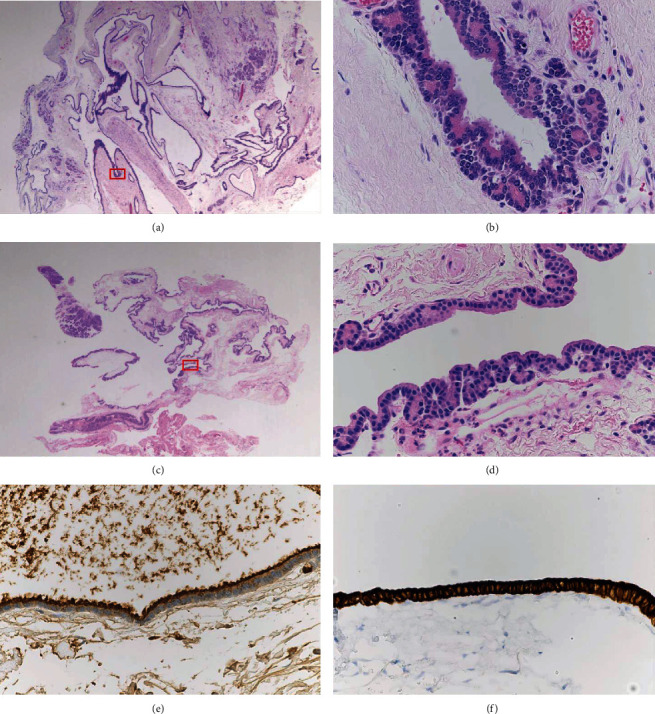
Microscopic images of ACT. Boxed areas are shown in a higher magnification on the right side of the corresponding picture. (a) ACT in the resection specimen consists of multiple variable-sized cysts (H&E, 20x magnification). (b) The lesion is lined by cells with acinar differentiation which have apical eosinophilic cytoplasmic granules and uniform nuclei. Cytologic atypia and mitotic figures are absent (H&E, 400x magnification). (c) Prior intraoperative biopsy of the cyst wall (H&E, 20x magnification). (d) The prior intraoperative biopsy shows similar epithelial lining to that seen in the resection specimen (H&E, 400x magnification). (e) The lining cells have coarsely granular apical cytoplasmic staining for trypsin (400x magnification). (f) The lining cells have strong and diffuse cytoplasmic staining for CK7 (400x magnification).

On gross examination, a multilocular cystic lesion was identified in the head of the pancreas with cysts ranging from 0.5 to 3 cm in size (Figure 2). The cysts were filled with clear fluid, and the cystic wall was smooth without any solid or papillary areas. Microscopically, the lesion consisted of multiple variable-sized cysts separated by fibrous stroma and residual islands of unremarkable pancreatic tissue (Figure 3(a)). The cysts contained eosinophilic secretions and were lined by a single layer of bland epithelium ranging from nondescript flat/cuboidal epithelium to apparent acinar cells (Figure 3(b)). Cytologic atypia and mitotic figures were absent. Occasional calcifications with fibrosis, histiocytes, and cholesterol clefts were noted. The immunohistochemical analysis was performed and demonstrated that the cyst lining cells were positive for trypsin (Figure 3(e)), CK7 (Figure 3(f)), and CK19 but negative for synaptophysin and chromogranin. Proliferative index marker (Ki-67) is less than 1%. Based on these histomorphological and immunohistochemical findings, the final diagnosis was confirmed to be ACT. The prior intraoperative biopsy was retrospectively reviewed to reveal similar epithelial lining to that seen in the resection specimen (Figures 3(c) and 3(d)). One and a half years have passed after surgical resection and no recurrences of the lesion and symptoms were documented.

## 3. Discussion

Pancreatic cystic lesions are increasingly identified in recent years due to the significant improvement of medical imaging technologies. According to the histopathology, pancreatic cystic lesions can be categorized into three main groups: pseudocyst, cystic neoplasms with epithelial linings, and cystic degeneration of solid neoplasms such as solid pseudopapillary neoplasm, neuroendocrine tumors, and ductal adenocarcinoma. The cystic neoplasms with epithelial linings include serous cystadenoma, mucinous cystic neoplasm (MCN), intraductal papillary neoplasm (IPMN), and acinar cystic transformation (ACT). Distinguishing between the various types of cystic pancreatic lesions has important prognostic and therapeutic implications.

Acinar cystic transformation (ACT) is an extremely rare benign pancreatic cystic neoplasm. Although it was firstly reported in 2002 as an incidental finding during autopsy [2], ACT was eventually recognized as a new entity by the World Health Organization (WHO) in 2010. To date, 25 papers including 75 patients were identified in the literature based on the most recent review article [26]. In the reported cases, ACT has a female predominance (female to male ratio: 2.4 : 1) with a wide age range (9–68 years). About two-thirds of the patients presented with abdominal pain. ACT was found in all parts of the pancreas with half of the cases located in the head of the pancreas, but there were two cases where ACT occurred outside the pancreas. ACT can be either unilocular or multilocular, and its sizes range from 0.5 to 19.7 cm.

The preoperative diagnosis of ACT based on imaging is very difficult due to the lack of specific radiologic features of ACT. The cytology results of fine-needle aspirations are also usually inconclusive because of the limited cellularity [24]. Due to those facts, the definitive diagnosis of ACT was usually established based on pathological examination of the resection specimens. Microscopically, ACT is lined by a single layer of bland flattened/cuboidal to normal apparent acinar cells with apical granular eosinophilic cytoplasm and basally located uniform nuclei. Ductal epithelium can be admixed with the acinar cells. Mitotic activity, cytologic atypia, necrosis, infiltrative growth, and ovarian-type stroma are absent. In immunohistochemical analysis, the cyst lining cells are positive for CK7, CAM 5.2, and acinar cell markers including trypsin, chymotrypsin, and lipase. CK19 stains intervening ductal epithelial cells. The Ki-67 index is less than 1%.

To date, the pathogenesis of ACT still remains unclear. Khor et al. identified multiple chromosomal gains involving 1p, 3p, 5q, 6p, 7q, 8, 10q, 11, 14, 20, and X in one case of multilocular ACT with mural nodules by using array comparative genomic hybridization, suggesting ACT could be a neoplastic lesion [9]. However, subsequent studies found a random X-chromosome inactivation pattern in 5 ACT cases and the lack of mutations in *KRAS*, *SMAD4*, *TP53*, and *beta-catenin* in 4 ACT cases without mural nodules, supporting that ACT may be a nonneoplastic dilation of the acinar and ductal epithelium [13, 25].

Although ACT is no longer thought to represent the benign counterpart to acinar cell cystadenocarcinoma, the true malignant potential of ACT has not yet been determined due to the rarity of this entity and relatively short periods of follow-up in the reported cases. Up to now, there are no reports of malignant transformation or metastasis of ACT with up to 10-year follow-up, regardless of whether the ACT was completely resected or not [26].

Here, we reported a case of ACT of the pancreas in a young female with 15-year follow-up. Our case report is unique in that it is the longest period of follow-up for ACT reported thus far in the literature. In our case, the patient had abdominal pain as first presentation and was found to have a cystic lesion in the region of pancreatic head on abdominal CT scan. During the exploratory laparotomy, the frozen section of an incisional biopsy was performed to confirm that the lesion was a true pancreatic cyst without malignancy. Due to the young age of the patient and lack of evidence of malignancy, the cystic lesion was treated with intraoperative drainage and unroofing. Then, our patient was placed under close ultrasound surveillance. For the next fifteen years, the patient did not experience any related symptoms or complications and the lesion grew from 4.2 cm to 6.2 cm in diameter (0.13 cm per year). In addition, the patient had a successful uncomplicated pregnancy during this period. Only in the latest 5 months, the patient started having occasional abdominal pain and discomfort. Then, a pylorus-preserving pancreaticoduodenectomy was performed due to her recurrent pain and enlargement of the lesion. The prior intraoperative biopsy slide was retrospectively reviewed and showed similar epithelial lining to that seen in the resection specimen, suggesting that ACT is a slow-growing neoplasm without malignant transformation after fifteen years.

Currently, a surgical resection is recommended for both diagnosis and treatment of ACT. However, complete resections including distal pancreatectomy with or without splenectomy, pancreaticoduodenectomy, and central pancreatectomy can associate with many postsurgical complications and high morbidity, especially in a young patient. Wang et al. reported in their case series of ACT that 5 out of 8 patients that received surgical resection had postsurgical complications including pancreatic fistula, gastroplegia, pneumonia, and intestinal obstruction [14]. In addition, pancreatectomy also increases patient's risk for uncontrollable diabetes, leading to increased long-term mortality [27]. Therefore, we recommend that the ACT should stay in the differential diagnosis of cystic lesions in the pancreatic head. To avoid complications of an extensive surgical procedure, the incisional biopsy, rather than surgical resection, may be a better option for asymptomatic or young patients with ACT. Intraoperative drainage and unroofing of ACT can be performed to relieve mass effect-related symptoms followed by close ultrasound surveillance. Surgical resection should be reserved for patients with persistent abdominal symptoms or suspected malignancy.
